# Restoring ion channel pathology by parameter optimization

**DOI:** 10.1186/1471-2202-12-S1-P334

**Published:** 2011-07-18

**Authors:** Jenny Tigerholm, Erik Fransén

**Affiliations:** 1 Department of Computational Biology, School of Computer Science and Communication, Royal Institute of Technology, Stockholm, Sweden; 2 Stockholm Brain Institute, Royal Institute of Technology, Stockholm, Sweden

## 

In diseases of the brain, the distribution and properties of ion channels display deviations from healthy control subjects. We studied two cases of ion channel alterations related to epileptogenesis. The first case of ion channel alteration represents an enhanced sodium current, the second case addresses the run down of conductance of the transient potassium current, K_A_. In previous studies we have shown that K_A_ reduce highly synchronized synaptic input while minimally affect semi-synchronized input (1). The K_A_ channel may therefore function as a protective mechanism against synchronous input involved in seizures. In this study we investigate if modulatory substances which targets the K_A_ can functionally corrected the two pathological models.

## Methods

We used a detailed compartmental model of a CA1 pyramidal cell based on a model published by Poolos et al 2002 (2). The model includes sodium, delayed rectifier potassium, A-type potassium and h channel models. On three medial distal dendrites 20 excitatory synaptic inputs were placed. The 20 synaptic inputs had a temporal distribution with different standard deviation corresponding to different levels of synchronicity. The modulation of K_A_ by two substances, KChIP1 and DPP6, was implemented in the model from experimental data. These substances produce shifts of activation, inactivation and time constant curves. Relative concentrations of these modulators were controlled by a numerical optimizer which compared model output to predefined neural output which represented a normal physiological response.

## Result

**Figure 1:**
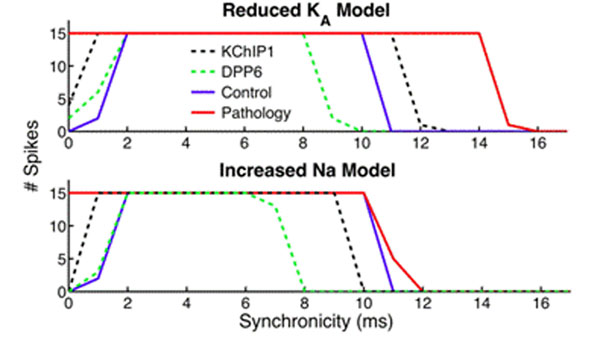


DPP6 and KChIP1 can functionally correct the two pathological models. Figure 1 shows spike activity during control conditions (blue) as well as spike activity for the pathological models (red). Top trace shows the spike activity produced by the reduced K_A_ model and the bottom increased Na model. Note the strong reduction is spike activity in the control case for highly synchronized input (0-2ms). In both the pathological models the K_A_ current is not strong enough to suppress highly synchronized input. For the functionally corrected pathological models (dotted lines), the example when KChIP1 was used as a free variable (black) and when DPP6 was used as the free variable (green) for the optimization, are shown.

## Conclusions

Our simulations show that an increase of KChIP1 or of DPP6 can functionally correct both pathological models’ response to different levels of synchronized input. These modulatory substances could be beneficial in reducing epileptic activity and could be candidates for drug development.
